# Activity-Dependent Changes in Cholinergic Innervation of the Mouse Olfactory Bulb

**DOI:** 10.1371/journal.pone.0025441

**Published:** 2011-10-28

**Authors:** Ernesto Salcedo, Tuan Tran, Xuan Ly, Robert Lopez, Cortney Barbica, Diego Restrepo, Sukumar Vijayaraghavan

**Affiliations:** 1 Department of Cell and Developmental Biology, University of Colorado, School of Medicine, Aurora, Colorado, United States of America; 2 Department of Physiology and Biophysics, University of Colorado, School of Medicine, Aurora, Colorado, United States of America; 3 Neuroscience Program, University of Colorado, School of Medicine, Aurora, Colorado, United States of America; Duke University, United States of America

## Abstract

The interplay between olfactory activity and cholinergic modulation remains to be fully understood. This report examines the pattern of cholinergic innervation throughout the murine main olfactory bulb across different developmental stages and in naris-occluded animals. To visualize the pattern of cholinergic innervation, we used a transgenic mouse model, which expresses a fusion of the microtubule-associated protein, tau, with green fluorescence protein (GFP) under the control of the choline acetyltransferase (ChAT) promoter. This tau-GFP fusion product allows for a remarkably vivid and clear visualization of cholinergic innervation in the main olfactory bulb (MOB). Interestingly, we find an uneven distribution of GFP label in the adult glomerular layer (GL), where anterior, medial, and lateral glomerular regions of the bulb receive relatively heavier cholinergic innervation than other regions. In contrast to previous reports, we find a marked change in the pattern of cholinergic innervation to the GL following unilateral naris occlusion between the ipsilateral and contralateral bulbs in adult animals.

## Introduction

The main olfactory bulb (MOB) receives numerous inputs from the rest of the brain that far exceed the number of bulb outputs. While these centrifugal inputs have been implicated in the modulation of olfactory behavior, their anatomical and functional significance remains unclear. From the basal forebrain, cholinergic neurons residing in the horizontal limb of the diagonal band of Broca (HDB) project varicose fibers in a diffuse pattern into the MOB [1]–[3]. These cholinergic projections can be observed across different MOB layers, with dense fiber expression in a small number of posterior “atypical” glomeruli [3]–[5].

In rodents, the density of cholinergic projections throughout all layers in the MOB increases in the first three weeks and decreases in the adult [3], [6]. This stratification of cholinergic innervation parallels the laminar distribution of cholinergic receptors [6]. With the exception of dense cholinergic innervation of a small number of atypical glomeruli, little is known about the spatial inhomogeneity of MOB cholinergic input throughout the MOB. In fact, previously demonstrated odor-to-odor differences on the effects of cholinergic pharmacological agents on olfactory behavior [7] may be due to inhomogeneity in cholinergic innervation or receptor expression.

Disruption of cholinergic modulation, mediated either by mAChR- or nAChR-specific agents, results in significant changes in spontaneous odor discrimination [8], perceptual learning [7], [9], and olfactory memory [10], [11]. Such changes implicate cholinergic modulation of the bulb in behavioral responses to odors. Further, the concurrent disruption in both the cholinergic innervation and olfaction early during neurodegenerative diseases like Alzheimer's disease [12], [13], suggests an important role for this centrifugal input.

While it is evident that the cholinergic innervation has profound effects on olfactory function (see above), it is not clear whether the innervation is, in turn, dependent on olfactory experience. Counter to what one might have predicted, it has been reported that olfactory input does not influence cholinergic innervation [14], though it significantly affects the noradrenergic [14], [15] and serotonergic [16] systems. Such a counterintuitive result may have been due to the choice of acetylcholine esterase (AChE) staining [14], which is less specific [17] as a marker for cholinergic fibers, and from the lack of stringent quantitation. To date, a quantitative estimation of changes in this centrifugal input throughout the bulb remains lacking.

We have developed a transgenic mouse model designed for the quantitative evaluation of spatial patterns of cholinergic centrifugal input, where expression of a tauGFP fusion protein is driven by the choline acetyl transferase (ChAT) promoter [18]. This model, due to cytoskeleton-associated GFP expression, allows for a much clearer resolution of the axon branches than ChAT immunostaining, presumably because of low axoplasmic volumes. Here, we use this model to examine cholinergic innervation patterns, in three dimensions, and in a quantitative manner. We show detailed spatial localization of cholinergic processes, developmental changes, and activity-dependent alterations in cholinergic innervation of the MOB. Our results show, for the first time, olfactory activity and experience-driven patterning of cholinergic innervation in the bulb.

## Results

### ChAT-GFP labeling is found throughout the MOB in adult animals

**Figure 1:**
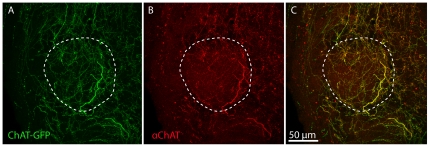
ChAT-GFP colabels with anti-ChAT antibody and distinctly labels cholinergic fibers with a greater signal to noise ratio so that finer details of the cholinergic fibers can be resolved. **A.** Single color channel (A; GFP & B; ChAT) and merged (C) confocal images of a glomerulus (dashed line) in the main olfactory bulb.

**Figure 2:**
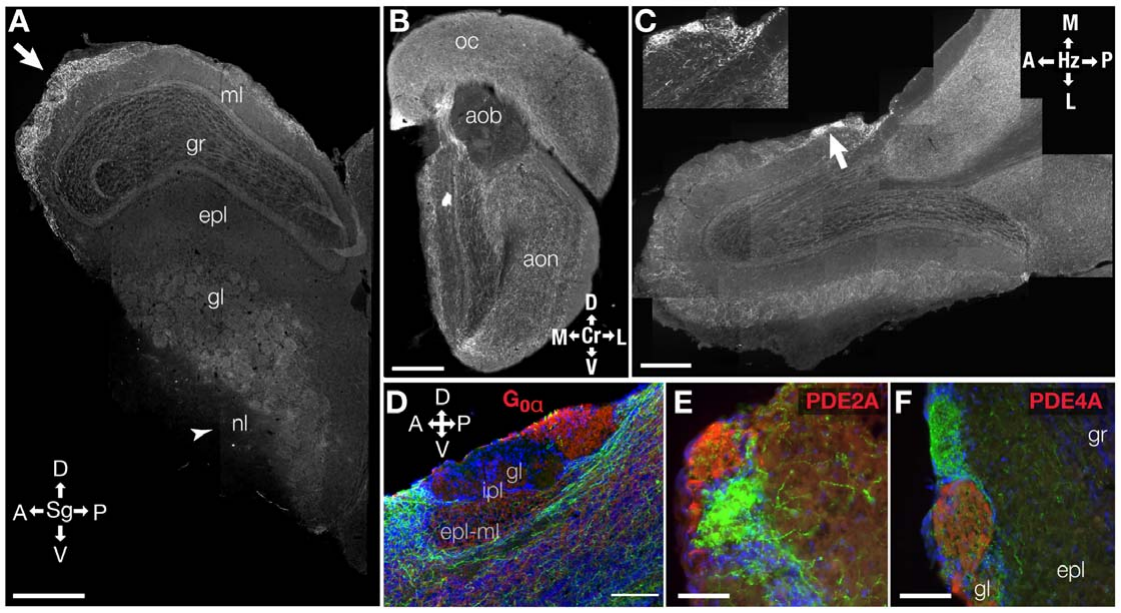
Micrographs of main olfactory tissue from Chat-GFP mice show diffuse labeling throughout the bulb with some regions of high labeling in the GL. ***A–C*** **.** High-resolution composite micrographs (shown for visualization only) of MOB tissue. Plane orientation: Sg - sagittal, Cr - coronal, Hz - horizontal; a = anterior, p = posterior, d = dorsal, v = ventral, l = lateral, m = medial. Layer labels: *nl -* nerve; *gl -* glomerular; *epl* - external plexiform; *gr* - granule cell; *ml* - mitral cell; *ipl* - internal plexiform. Scale bar indicates 500 µm. ***A.*** Parasagittal section. *Arrow* points to region of relatively heavy GFP labeling in the anterior region of the bulb. *Arrow head* indicates region where relatively little labeling is found. ***B.*** Coronal section. *oc -* orbital cortex. *aob* - accessory olfactory bulb. *aon -* anterior olfactory nucleus. ***C.*** Micrograph of a coronal cross-section of the MOB and AOB. Arrow points to heavily staining glomeruli shown in inset. ***C inset*** **.** High-resolution micrograph of glomeruli with a relatively high amount of GFP staining. ***D*** **.** High-resolution micrograph of the AOB showing that the relatively light GFP staining in the anterior portion of the structure did not co-label with G_oα_ labeled in red. ***E–F*** **.** Heavily-stained GFP glomeruli do not co-label with PDE 2A (**E**) or PDE 4A (**F**). Red = PDE#A, Green = GFP. Scale bar = 100 µm.

As shown in Figure 1, the ChAT-GFP label is a robust marker of cholinergic fibers that colabels with the anti-ChAT antibody while at the same time providing far greater resolution and detail. Using the ChAT-GFP label, we found little evidence of cholinergic interneurons in the bulb; however, we did find substantial tauGFP labeling of cholinergic processes throughout the MOB (Figure 2A–C) in a pattern similar to those previously reported in the rodent main olfactory bulb using ChAT immunohistochemical studies [3]. While the staining intensity appeared heterogeneous throughout most layers of the MOB, we found regions of comparatively high intensity GFP staining in the GL, including the heavily stained glomeruli previously identified and termed ‘atypical’ based on acetylcholinesterase (AChE) histochemistry [4], [19], [20]. Consistent with previous reports, the heavily labeled glomeruli lay adjacent to “necklace glomeruli” stained with PDE 2A [21] or other glomeruli stained with PDE 4A [21]–[23]. As expected, we found little expression in the nerve layer of the MOB (Figure 2A, arrowhead). Whereas dense process labeling was seen adjacent to the accessory olfactory bulb (AOB), the structure itself was sparsely innervated. Within the AOB, however, there was heterogeneity in the innervation, with the fiber expression and the expression of G_oα_ being mutually exclusive (Figure 2B, 2D).

### ChAT-GFP innervation in the GL changes throughout postnatal development from relatively homogeneous to inhomogeneous

**Figure 3:**
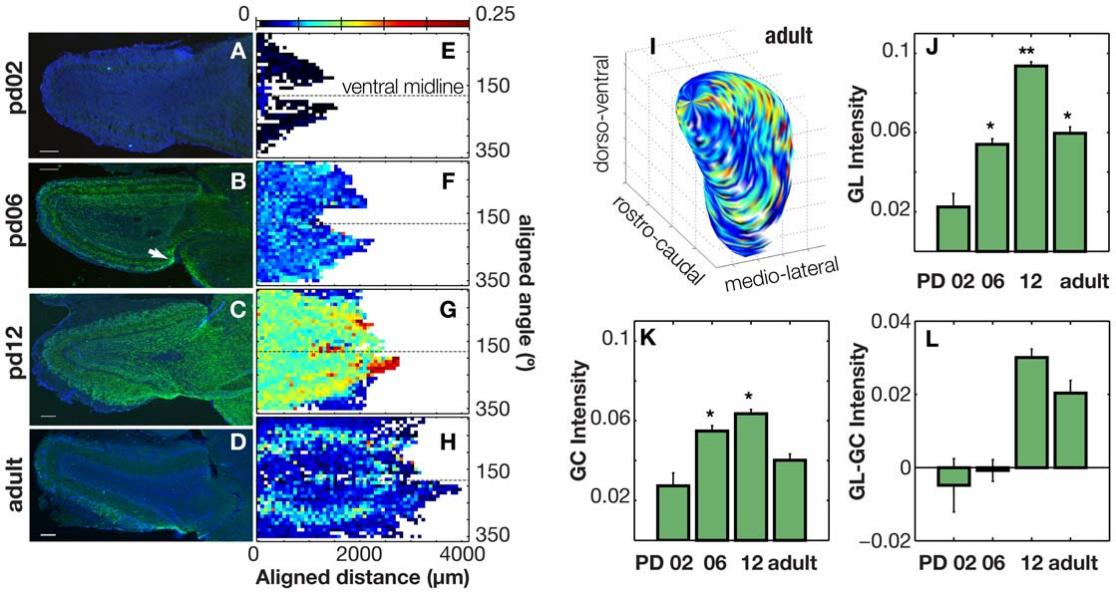
The pattern and intensity of GFP staining in the GL and GCL of the MOB across different developmental stages in ChAT-GFP mice. **A–D.** Representative cross-sections of MOB cut horizontally from four different age groups: Post-natal day (PD) 02, 06, 12, and adult. Scale bar indicates 100 µm. **E–H** Average intensity maps of GFP-staining across the glomerular surface of each MOB. *Abscissa*: rostro-caudal position realigned to the standard bulb where 0 µm corresponds to the anterior end of the bulb. *Ordinate:* cylindrical coordinates realigned so that 0° corresponds to dorsal, 90° to lateral, 180° to ventral, 270° to medial surfaces of the bulb. The color bar indicates the level of corrected green intensity in the GL of the MOB with warm colors indicating higher levels of green intensity. **I.** Intensity map from H overlaid on a standardized, three-dimensional representation of the inner GL of the MOB showing the heterogenous staining pattern on the lateral surface of the bulb. Axes range: d – dorsal, v- ventral; r – rostral, c – caudal; m – medial, l – lateral. **J–L.** Changes in GFP intensity in the glomerular (GL) and granule cell (GC) layers of the bulb across age groups. **J.** The level of GFP intensity in the **GL layer** significantly increases up to post-natal day (PD) 12 and then decreases in adult animals (Anova p-value: 3×10^−36^). A multiple comparison test using Tukey's HSD criterion shows: ** PD12 mean GL intensity is significantly greater than all other age groups. * PD06 and Adult mean GL intensities are significantly greater than PD02, but significantly lower than PD12. They are not significantly different from each other. **K.** The level of GFP intensity in the **GC layer** significantly levels out at PD6 and then decreases in adult animals (Anova p-value: 3×10^−36^). L. GC intensity subtracted from GL intensity highlighting the change in relative intensity between GC and GL during development.

A comprehensive examination of the glomerular layer revealed a changing pattern of cholinergic innervation throughout the MOB across different age groups. We found very little GFP labeling at postnatal day 2 (PD 02), but the label was discernible at PD 06. GFP label showed a more uniform increase by PD 12, and then a heterogeneous decrease in adult animals (Figure 3A–D). These changes occurred throughout the extent of the bulb and in different animals (Figure 3E–K). Heavily labeled, atypical glomeruli were seen as early as PD 06 (Figure 3B, arrow). To correlate GFP intensity with the extent of cholinergic innervation in a given glomerular region of interest (ROI), we calculated a positive pixel ratio (see methods) to determine the number of pixels in a given ROI that had a calculated intensity that was greater than background intensity. This ratio showed a significant positive correlation to GFP intensity for all age groups (r = 0.5432, 0.3555, 0.2656, 0.3585, from PD 02 to adult; p<1×10^−55^ for all ages; Pearson). As each pixel represents a defined unit of area in the imaged tissue, the level of GFP intensity therefore positively correlates to the area of tissue labeled with GFP (as opposed to a small, uniform number of pixels simply growing brighter). The relatively low r-values highlights the level of variability in GFP intensity that we see at a given positive pixel ratio, particularly as the positive pixel ratio approaches one. Since these images were taken using a standard epifluorescence microscope, such variability may be indicative of underlying changes in cholinergic density throughout the volume of the cryosection that is not evident in the two-dimensional image. Regardless, we visually confirmed the correlation by calculating the intensities and positive pixel ratios of heavily labeled atypical glomeruli. These glomeruli consistently had both a greater GFP intensity and a higher positive pixel ratio than their neighboring, less innervated glomeruli (data not shown).

**Figure 4:**
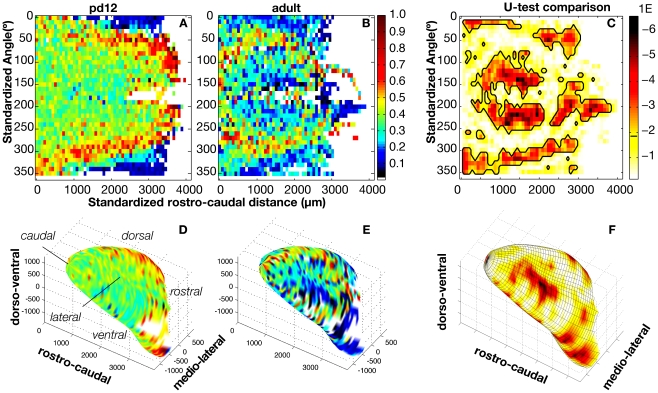
Standardized and normalized GFP intensity maps show significant differences in the distribution of GFP intensity between PD12 and adult age MOBs. **A–B.** GFP intensity maps realigned to standardized rostro-caudal and dorso-ventral positions. **C.** A map of p-values generated from a bin-wise Mann-Whitney U test comparing bins with identical cylindrical coordinates across the PD 12 and adult GFP intensity maps. Each p-value is calculated from a *U*-test comparing bins from intensity maps across both occluded and non-occluded bulbs that have identical cylindrical coordinates. p-values that fall below the false discovery rate (0.014, hatch on the color bar) are circumscribed in black and indicate regions on the map that exhibit a statistically significant change in the level of GFP intensity **D–F.** Probability map from **A–C** overlaid on a 3D reconstruction of the olfactory bulb (standard bulb). Wire mesh in F indicates the bins compared in C.

To compare GFP intensity in different layers of the MOB, we examined the mean GFP intensities from the glomerular layer (GL) and the granule cell layer (GCL) (Figure 3J–K) in each bulb. Whereas PD 02 and PD 06 MOBs had relatively greater GFP intensity in GCL than in the GL, PD 12 and adult bulbs had greater GFP intensity in the GL than in the GCL (Figure 3L). GFP intensity in the GL appeared to increase homogeneously throughout the bulb in the PD 02, PD 06, and PD 12 animals (a five-fold increase from P02 to P12) and then decreased heterogeneously (Figure 3E–I), resulting in patterns of high GFP intensity in the medial and lateral aspects seen in the adult bulb (Figure 2). This suggests that the intensity pattern seen in the adult bulb arises from the loss of cholinergic innervation that occurs during normal development of the MOB. To characterize the nature of this pattern in relation to overall GFP intensity, we normalized intensity to the maximum intensity recorded per MOB, thereby controlling for animal-to-animal variation in the intensity of GFP labeling. We also controlled for inter-animal variation in bulb size by scaling each bulb to match the standard bulb and then plotting the mean intensity maps in standardized space (see Materials and Methods). The resultant map of GFP intensity for PD 12 and adult maps (Figure 4A, 4B) resemble the non-normalized maps (Figure 3G, 3H), suggesting that the patterns of intensity are independent of the overall level of intensity and size of a given bulb. Moreover, a bin-wise Mann-Whitney U test of these normalized intensity maps revealed regions of significant difference in intensity values along the ventro-lateral, ventro-medial, and dorsal aspects of the bulb (Figure 4C - *regions circumscribed in black*). The location of these regions of significant differences are more easily discerned in the 3D representation of the bulb; e.g. the large region colored in red (or warm colors) on the dorso-lateral surface of the MOB in Figure 4D.

### Naris occlusion abolishes localized immunostaining of high GFP intensity in the GL of adult bulbs

**Figure 5:**
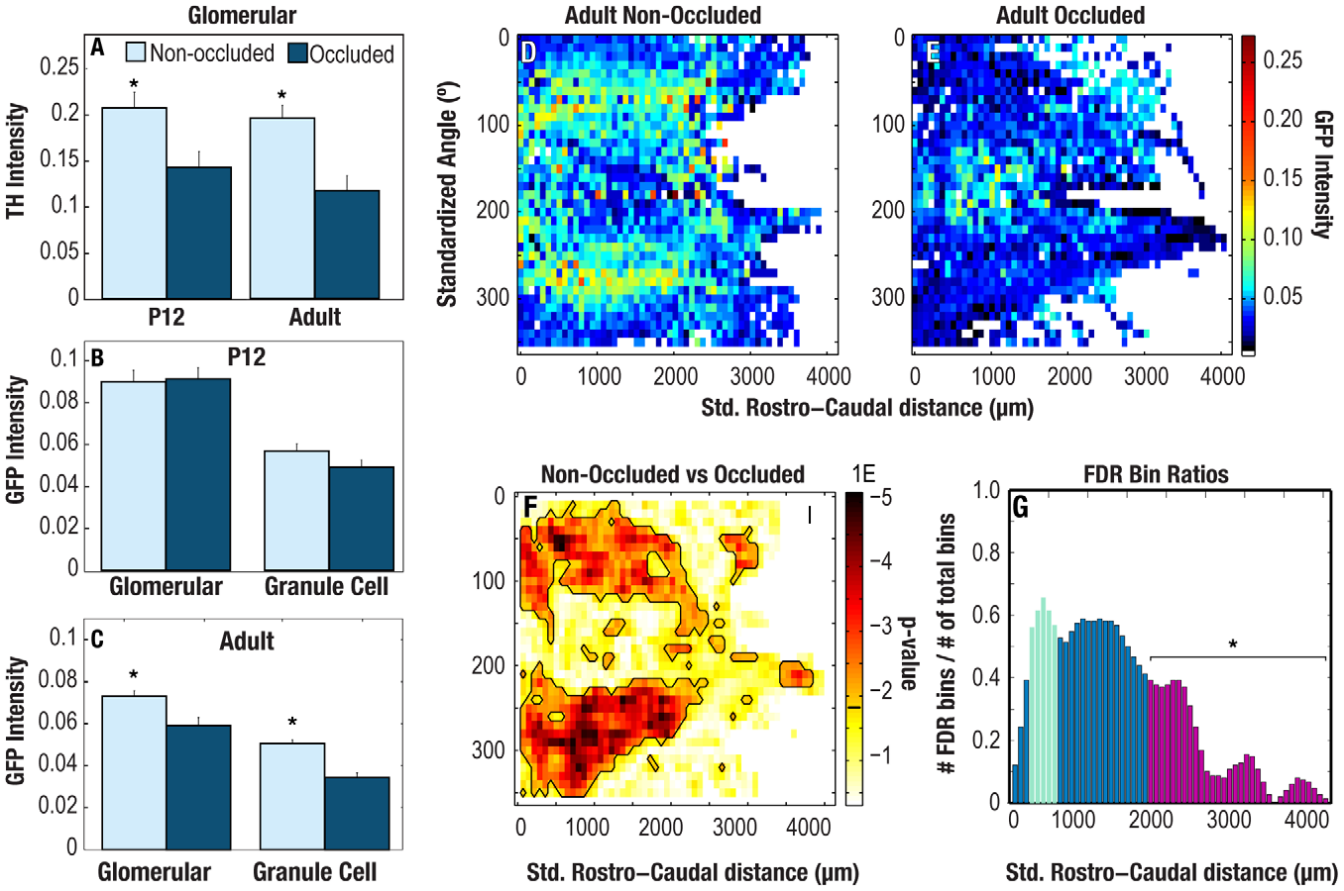
Naris occlusion abolishes differential GFP staining pattern in adult animals. **A.** Significantly lower TH Intensity in occluded bulbs as compared to non-occluded bulbs confirmed that the occluded bulbs had reduced olfactory activity in both PD12 animals and adult animals (*t*-test, *p*-value = 1×10^−2^ and 3.6×10^−4^, respectively). **B.** PD12 animals: mean GFP intensity did not vary significantly between occluded and non-occluded bulbs in either the glomerular or granule cell layers of PD12 bulbs. **C.** Adult animals: mean GFP intensity fell significantly in both the glomerular and granule cell layers in the occluded bulbs of adult animals (*t*-test, *p*-values: 2.2×10^−3^ and 2.4×10^−8^, respectively). **D–E.** Average intensity map in the occluded bulb (E) showed a markedly reduced differential patterning of GFP intensity throughout the GL as compared to the non-occluded bulb. **F.** p-value map generated from a binwise Mann-Whitney U test where p-value is indicated by color. Statistically significant p-values that fall below the false discovery rate (0.02, as indicated by the black line on the color bar) are circumscribed in black. These circumscribed bins indicate regions of the glomerular layer that differ significantly in GFP intensity between the occluded and non-occluded bulbs. **G.** The ratio of statistically significant bins per column of positive bins in the *U*-test map increases from the caudal end of the bulb towards the rostral end as discussed in the text. Magenta- colored bars (indicated by * line) in the bar plot differ significantly from the mean of the green bars (paired-ratio *t*-test, corrected for multiple comparisons using the false discovery rate, p-value = 0.0071).

To determine the effect that olfactory sensory neuron activity has on the heterogeneous pattern of GFP staining that we found in the adult MOBs, we occluded one naris at PD 02. We compared tyrosine hydroxylase (TH) and GFP immunostaining intensity in the GL and GCL from PD 12 and adult olfactory bulbs ipsilateral and contralateral to the occluded naris. TH has previously been shown to be an effective reporter of odor-modulated afferent activity in the bulb [15]. As expected, we found a significant decrease in the mean intensity of TH immunolabel in the GL ipsilateral to the occluded naris as compared to the corresponding contralateral layer in both the PD 12 and adult bulbs (Figure 5A). In contrast, we detected no difference in the overall mean GFP intensity in the GL or GCL of the contra- or ipsi-lateral bulbs in PD 12 animals (Figure 5B). This could either be due to the lack of an effect of afferent activity on cholinergic innervation of the MOB during early development or to the shorter time that the naris is occluded in PD 12 mice than in the adults. However, we did find a significant decrease in the overall mean GFP intensity in the GL and GCL of the bulb ipsilateral to the occluded naris in the adult bulb as compared to the same layers in the contralateral bulbs (Figure 5C). More importantly, we also found that the localized pattern of high GFP intensity along the medial and lateral aspects of the bulb is abolished in the GL ipsilateral to the occluded naris (Figure 5D–F). These results indicate that OSN activity is critical for maintaining a high level of cholinergic innervation to localized regions of the GL in adult bulbs. Interestingly, the effect of naris occlusion appeared diminished in the posterior region of the bulb as seen Figure 5E (magenta bars), in which we counted a reduced number of bins with a significant difference in GFP intensity between the occluded and non-occluded bulbs. Such a result is in line with previous studies indicating that OSNs, which target the posterior olfactory bulb, could potentially receive access to odors via retronasal or internasal sources [24], [25].

## Discussion

In this study, we employ a transgenic mouse model that allows for clear resolution and quantification of changes in the spatial pattern of GL innervation by cholinergic fibers. Using this model, we find that the spatial pattern of cholinergic innervation of the GL changes from being homogeneous up to PD 12 to inhomogeneous in the adult mouse. In addition, we find that naris occlusion disrupts this inhomogeneous pattern in the GL of adult mice. Our study represents the first quantitative characterization of cholinergic innervation to the mammalian olfactory bulb. Counter to previous reports, we show that olfactory experience sculpts the pattern of- cholinergic innervation from the basal forebrain.

As seen in Figure 1 and in Grybko et al. [18], our model shows a high degree of fidelity in labeling cholinergic processes. Moreover, the distribution of these cholinergic processes throughout the MOB corresponds with previous studies in mouse, rat, and monkey models [26], [27] that show the GL in adult MOBs receives the highest level of cholinergic innervation and has higher levels of presynaptic cholinergic drug binding sites [6]. These results suggest that the changes we observe are mediated by alterations in innervation, rather than a change in the transgene expression. The highest levels of cholinergic fiber density seen in the ventro-anterior and ventro-medial portion of the bulbs in our mice have previously been reported and termed ‘atypical’ glomeruli [3] . Our data demonstrate that these heavily innervated glomeruli are adjacent to, but exclude, the ‘necklace glomeruli’ described in the MOB [21], [22], suggesting that the cholinergic system might not selectively modulate the guanylate cyclase-dependent transduction mechanisms in the bulb. However, the physiological role of these atypical glomeruli remains to be determined.

Cholinergic innervation of the AOB appears relatively sparse with a large portion of incoming GFP-positive fibers circumventing the structure. Interestingly, those fibers that do innervate the AOB preferentially target the anterior aspect of this structure and do not co-label with G_oα_. This pattern is consistent with the distribution of vomeronasal projections expressing G_iα_ [28]; however, the question of whether cholinergic modulation of AOB processing is differentially regulated remains to be examined. Interestingly, exogenous activation of cholinergic receptors control the excitability of mitral cells in the AOB [29].

During development, cholinergic projects increasingly target the bulb, starting after PD 02 and reaching maximal levels by PD 12. Initially, the pattern of innervation is fairly homogenous and appears evenly distributed throughout the bulb; however, by adulthood, the pattern becomes spatially organized, with the GL receiving a higher level of innervation in comparison to the rest of the bulb. This differential distribution of laminar innervation suggests large-scale axonal pruning, but whether this pruning reflects axon elimination or apoptotic loss of HDB cholinergic neurons remains to be investigated. The differential pattern of cholinergic innervation seen within the GL itself implies interplay between olfactory activity and cholinergic modulation. The identification of odorant specificities of atypical glomeruli will help elucidate behavioral significance of this modulation.

Cholinergic mechanisms are important in modulating stereotypic patterning of innervation. In fact, nicotinic acetylcholine receptors play an important role in activity-dependent patterning in the mammalian retina [30], [31]. While modulation of primary networks by cholinergic mechanisms has been examined in recent years, much less is known regarding activity-dependent sculpting of cholinergic innervation itself. At the neuromuscular junction, muscle activity regulates the number of surviving motoneurons [32]. In the retina, spatial distribution of cholinergic amacrine cells and their connectivity is dependent on visual activity [33]. The role that olfactory activity plays in the pruning/elimination of cholinergic projections to the bulb remains to be determined. As changes in the patterning and survival of cholinergic projections might underlie the etiology of a number of neurodegenerative diseases, the olfactory bulb provides a useful model to examine the mechanistic underpinnings of these processes.

## Materials and Methods

### Animals

We generated a bacterial artificial chromosome (BAC) containing a fusion of the microtubule-associated protein, tau, with green fluorescence protein (GFP) under the control of the choline acetyltransferase (ChAT) promoter [18]. Breeding was performed at the transgenic facility at the Center for Comparative Medicine, University of Colorado, School of Medicine. Mice were group-housed in Micro-VENT cages (MBS75JHTMV) from Allentown Inc (Allentown, NJ) with complete air exchange at a rate of 40 times per hour. Water and food were made available *ad libitum*. Soiled litter was replaced every two weeks. All animals used were sacrificed according to institutional guidelines. For all of the experiments listed, we used a total of 20 ChAT-GFP mice, 10 females and 10 males.

### Ethics Statement

This study was carried out in strict accordance with the recommendations in the Guide for the Care and Use of Laboratory Animals of the National Institutes of Health. The protocol was approved by the Institutional Animal Care and Use Committee (IACUC) at the University of Colorado, School of Medicine. The approved IACUC Protocol Number is B-39609(05)1E. All efforts were made to minimize suffering.

### Immunohistochemistry

Mice were anesthetized with ketamine/xylazine (100 and 20 µg/g body weight, respectively), perfused transcardially with 0.1 M phosphate buffer (PB) followed by a PB buffered fixative containing 3% paraformaldehyde, 0.019 M L-lysine monohydrochloride, and 0.23% sodium m-periodate. Bulbs were harvested and postfixed for 2 hours. 18 µm transverse sections were cut using a cryostat, mounted on Superfrost plus slides (Fisher Science, Pittsburgh, PA), and stored in a −80°C freezer until used. Sections were rinsed and incubated in blocking solution containing 2% normal donkey serum, 0.3% Triton X-100, and 1% bovine serum albumin in PBS for 1.5 h. Sections were incubated for 72 h with primary antibodies against ChAT (1∶200, Millipore AB144P lot JC1618187), green fluorescent protein (GFP, 1∶2000, ab13970, Abcam, Cambridge, MA), tyrosine hydroxylase (TH, 1∶1000, gift from Dr. Michael Browning), Goα (1∶500, Millipore 07-634 DAM1614929, Billerica, MA), phosphodiesterase 2A (PDE2A, 1∶100 Santa Cruz, sc-17228 lot E1704, Santa Cruz, CA) and PDE4A (1∶1000, gift from Dr. James Cherry). After incubation of the primary antibodies, sections were washed and reacted with secondary antibody (1∶400, Alexa 488 or Alexa 568, Molecular Probes, Eugene, OR) for 1 hour at room temperature. Sections were mounted on slides with Fluoromount-G.

### Imaging

Confocal images were captured using an Olympus Fluoview confocal laser-scanning microscope (LSCM) FV300 (Olympus Corporation). To avoid any problems resultant from side-band excitation of the fluorochromes, channels in each image were collected sequentially with single wavelength excitation and then merged to produce the composite image using the Fluoview v5.0 software. Brightness and contrast were adjusted in Adobe Photoshop. The images of cryosections used for the intensity map reconstructions were digitally captured using Nikon Elements software driving a Nikon (Tokyo, Japan) Eclipse E600 microscope equipped with epi-fluorescence and a Nikon DS Qi1Mc camera.

### Mapping and Standard Bulb Fittings

To map the MOBs, we used our GLOM•MAP mapping software toolbox running in MATLAB (The MathWorks, Natick, MA) introduced in Salcedo et al. [34]. All bulbs used to generate the intensity maps were cryosectioned horizontally and each cryosection was imaged in full at 4×. We only mapped every fourth micrograph so that the spacing between micrographs was 72 µm along the dorsoventral axis. On each mapped micrograph, we segmented the boundary between the GL and the external plexiform layer (GL-EPL boundary) and the mitral cell layer. To sample intensity values at regular intervals, we applied adjacent circular regions-of-interest (ROIs) of varying radii throughout the entire GL, covering as much of the layer as possible. To establish a coordinate axes for each glomerular ROI, we manually aligned the GL-EPL boundary segments to a previously generated standard bulb in three-dimensional space. This standard bulb represents a digital reconstruction of the GL-EPL boundary from sixteen adult C57BL/6 mouse MOB [34] and consists of a collection of nodes, each with cylindrical coordinates r, ϕ and z. Steps for these nodes are 10 degrees for ϕ and 18 µm for z and have an associated distance within the lattice to an optimal location. During the alignment process, the mutual positions of the mapped point inside each section and mutual distances between sections were preserved. Once aligned, the angular coordinates of each ROI were recalculated so that 0°, 90°, 180°, and 270° correspond to the dorsal, lateral, ventral, and medial surfaces of the MOB, respectively (‘Aligned angle’ in the figures). Rostrocaudal distance was measured in reference to the caudal point of the standard bulb (“Aligned distance”). To compare maps across age groups and treatment groups, we re-calibrated the cylindrical coordinates for all ROIs by scaling the GL-EPL boundary of each bulb to account for individual bulb size differences, enlarging or shrinking each bulb along the three axes of the model to best match the size the standard bulb. These coordinates are presented as “Standardized Angle” and “Standardized Distance.”

### Intensity Calculations and Maps

We corrected all images for uneven illumination across the image field by normalizing to a standard background illumination image. We corrected for background illumination resulting from auto-fluorescence of the tissue by subtracting the background illumination from all intensity values in the image channel. Observing that the mitral cell layer of any given cryosection had a relatively low level of green intensity through the MOB, we defined background illumination as the mean intensity value from all pixels in this layer. To generate the intensity maps, we binned the circular glomerular ROIs by their cylindrical coordinates into 10° and 72 µm bins and then averaged the corresponding intensity values in each bin. Bins that contained no ROIs were assigned the value “NaN” (not-a-number) and were not used in statistical computations. For average maps, we averaged the intensity values from corresponding bins across all maps into a given statistical group.

### Positive pixel ratios

For each glomerular ROI, we counted the number of pixels in the ROI that had an intensity value above the background threshold and divided that number by the total number of pixels in the ROI. Thus, a positive pixel ratio of 1 indicates that 100% of the pixels in the ROI had an intensity that was greater than the background threshold.

### Naris Occlusion

Naris occlusion was performed as described [35]. This treatment is unlikely to affect the number of cholinergic neurons in HDB because harsher treatment such as unilateral transfection separating the olfactory bulb from the forebrain has not been described to affect the number of HDB neurons [36].

### Statistics

We used the Statistics Toolbox for MATLAB to perform all of the statistics and generate the intensity maps and 3D maps presented.

## References

[1] Macrides F, Davis BJ, Youngs WM, Nadi NS, Margolis FL (1981). Cholinergic and catecholaminergic afferents to the olfactory bulb in the hamster: a neuroanatomical, biochemical, and histochemical investigation.. J Comp Neurol.

[2] Zaborszky L, Heimer L, Eckenstein F, Leranth C (1986). GABAergic input to cholinergic forebrain neurons: an ultrastructural study using retrograde tracing of HRP and double immunolabeling.. J Comp Neurol.

[3] Durand M, Coronas V, Jourdan F, Quirion R (1998). Developmental and aging aspects of the cholinergic innervation of the olfactory bulb.. Int J Dev Neurosci.

[4] Zheng LM, Ravel N, Jourdan F (1987). Topography of centrifugal acetylcholinesterase-positive fibres in the olfactory bulb of the rat: evidence for original projections in atypical glomeruli.. Neuroscience.

[5] Le Jeune H, Jourdan F (1991). Postnatal development of cholinergic markers in the rat olfactory bulb: a histochemical and immunocytochemical study.. J Comp Neurol.

[6] Le Jeune H, Aubert I, Jourdan F, Quirion R (1996). Developmental profiles of various cholinergic markers in the rat main olfactory bulb using quantitative autoradiography.. J Comp Neurol.

[7] Hellier JL, Arevalo NL, Blatner MJ, Dang AK, Clevenger AC (2010). Olfactory discrimination varies in mice with different levels of alpha7-nicotinic acetylcholine receptor expression.. Brain Res.

[8] Mandairon N, Ferretti CJ, Stack CM, Rubin DB, Cleland TA (2006). Cholinergic modulation in the olfactory bulb influences spontaneous olfactory discrimination in adult rats.. Eur J Neurosci.

[9] Wilson DA, Fletcher ML, Sullivan RM (2004). Acetylcholine and olfactory perceptual learning.. Learn Mem.

[10] Ravel N, Elaagouby A, Gervais R (1994). Scopolamine injection into the olfactory bulb impairs short-term olfactory memory in rats.. Behav Neurosci.

[11] Rushforth SL, Allison C, Wonnacott S, Shoaib M (2010). Subtype-selective nicotinic agonists enhance olfactory working memory in normal rats: a novel use of the odour span task.. Neurosci Lett.

[12] Coyle JT, Price DL, DeLong MR (1983). Alzheimer's disease: a disorder of cortical cholinergic innervation.. Science.

[13] Christen-Zaech S, Kraftsik R, Pillevuit O, Kiraly M, Martins R (2003). Early olfactory involvement in Alzheimer's disease.. Can J Neurol Sci.

[14] Gomez C, Brinon JG, Colado MI, Orio L, Vidal M (2006). Differential effects of unilateral olfactory deprivation on noradrenergic and cholinergic systems in the main olfactory bulb of the rat.. Neuroscience.

[15] Baker H, Morel K, Stone DM, Maruniak JA (1993). Adult naris closure profoundly reduces tyrosine hydroxylase expression in mouse olfactory bulb.. Brain Res.

[16] Gomez C, Brinon JG, Orio L, Colado MI, Lawrence AJ (2007). Changes in the serotonergic system in the main olfactory bulb of rats unilaterally deprived from birth to adulthood.. J Neurochem.

[17] Crespo C, Brinon JG, Porteros A, Arevalo R, Rico B (1999). Distribution of acetylcholinesterase and choline acetyltransferase in the main and accessory olfactory bulbs of the hedgehog (Erinaceus europaeus).. J Comp Neurol.

[18] Grybko MJ, Hahm ET, Perrine W, Parnes JA, Chick WS (2011). A transgenic mouse model reveals fast nicotinic transmission in hippocampal pyramidal neurons.. Eur J Neurosci.

[19] Chen X, Wang L, Zhou Y, Zheng LH, Zhou Z (2005). “Kiss-and-run” glutamate secretion in cultured and freshly isolated rat hippocampal astrocytes.. J Neurosci.

[20] Gomez C, Brinon JG, Barbado MV, Weruaga E, Valero J (2005). Heterogeneous targeting of centrifugal inputs to the glomerular layer of the main olfactory bulb.. J Chem Neuroanat.

[21] Juilfs DM, Fulle HJ, Zhao AZ, Houslay MD, Garbers DL (1997). A subset of olfactory neurons that selectively express cGMP-stimulated phosphodiesterase (PDE2) and guanylyl cyclase-D define a unique olfactory signal transduction pathway.. Proc Natl Acad Sci U S A.

[22] Shinoda K, Ohtsuki T, Nagano M, Okumura T (1993). A possible functional necklace formed by placental antigen X-P2-immunoreactive and intensely acetylcholinesterase-reactive (PAX/IAE) glomerular complexes in the rat olfactory bulb.. Brain Res.

[23] Cherry JA, Davis RL (1999). Cyclic AMP phosphodiesterases are localized in regions of the mouse brain associated with reinforcement, movement, and affect.. J Comp Neurol.

[24] Coppola DM, Coltrane JA, Arsov I (1994). Retronasal or internasal olfaction can mediate odor-guided behaviors in newborn mice.. Physiol Behav.

[25] Royet JP, Jourdan F, Ploye H, Souchier C (1989). Morphometric modifications associated with early sensory experience in the rat olfactory bulb: II. Stereological study of the population of olfactory glomeruli.. J Comp Neurol.

[26] Kasa P (1986). The cholinergic systems in brain and spinal cord.. Prog Neurobiol.

[27] Porteros A, Gomez C, Valero J, Calvo-Baltanas F, Alonso JR (2007). Chemical organization of the macaque monkey olfactory bulb: III. Distribution of cholinergic markers.. J Comp Neurol.

[28] Halpern M, Martinez-Marcos A (2003). Structure and function of the vomeronasal system: an update.. Prog Neurobiol.

[29] Smith RS, Araneda RC (2010). Cholinergic modulation of neuronal excitability in the accessory olfactory bulb.. J Neurophysiol.

[30] Bansal A, Singer JH, Hwang BJ, Xu W, Beaudet A (2000). Mice lacking specific nicotinic acetylcholine receptor subunits exhibit dramatically altered spontaneous activity patterns and reveal a limited role for retinal waves in forming ON and OFF circuits in the inner retina.. J Neurosci.

[31] Feller MB, Wellis DP, Stellwagen D, Werblin FS, Shatz CJ (1996). Requirement for cholinergic synaptic transmission in the propagation of spontaneous retinal waves.. Science.

[32] Oppenheim RW, Prevette D, D'Costa A, Wang S, Houenou LJ (2000). Reduction of neuromuscular activity is required for the rescue of motoneurons from naturally occurring cell death by nicotinic-blocking agents.. J Neurosci.

[33] Zhang J, Yang Z, Wu SM (2005). Development of cholinergic amacrine cells is visual activity-dependent in the postnatal mouse retina.. J Comp Neurol.

[34] Salcedo E, Zhang C, Kronberg E, Restrepo D (2005). Analysis of training-induced changes in ethyl acetate odor maps using a new computational tool to map the glomerular layer of the olfactory bulb.. Chem Senses.

[35] Stone DM, Wessel T, Joh TH, Baker H (1990). Decrease in tyrosine hydroxylase, but not aromatic L-amino acid decarboxylase, messenger RNA in rat olfactory bulb following neonatal, unilateral odor deprivation.. Brain Res Mol Brain Res.

[36] Weiser M, Baker H, Joh TH (1994). Gene expression in central cholinergic neurons in response to axotomy and deafferentation.. Synapse.

